# Histopathological study using computer database of 10 000 consecutive gastric specimens: (2) malignant lesions

**DOI:** 10.1093/gastro/gou094

**Published:** 2015-02-09

**Authors:** Tadashi Terada

**Affiliations:** Department of Pathology, Shizuoka City Shimizu Hospital, Shizuoka, Japan

**Keywords:** stomach, malignant lesions, histopathology

## Abstract

Using a computer database, the author investigated the histopathology of 10 000 consecutive gastric specimens collected in the last 12 years 2002–2013 at his pathology laboratory in a relatively large hospital in Japan. Examination of histological sections was done when appropriate. The gastric specimens were made up of 8579 benign conditions and 1421 malignant lesions. The latter comprised gastric carcinoma in 1342 cases (94.4%), gastrointestinal stromal tumor (GIST) in 34 (2.4%), mucosal-associated lymphoid tissue (MALT) lymphoma in 25 (1.8%), non-Hodgkin's malignant lymphoma in 19 (1.3%), and metastatic carcinoma in 1 case (0.1%). Of the 1342 cases of gastric carcinoma, the histological type was as follows: tubular adenocarcinoma in 755 cases, papillary adenocarcinoma in 176, mucinous adenocarcinoma in 147, signet ring cell carcinoma in 145, poorly differentiated adenocarcinoma in 114, adenosquamous carcinoma in 4, and metastatic small cell carcinoma from the lung in 1. In surgically resected cases, the number of early gastric carcinomas was 258 and of advanced carcinoma, 521 cases. In GIST (*n = *34), there were 2 cases of the epithelioid type and 32 of the spindle cell type. The size of GIST ranged from 1–15 cm, with a mean of 5.6 cm. KIT (CD117) was positive in 34/34 cases, CD34 in 31/34, desmin 2/34, and S100 4/34. A genetic analysis was performed in 6 cases of GIST, all of which showed point mutation of KIT and/or PDGFRA genes. In MALT lymphoma (*n = *25), centrocyte-like cells and lymphoepithelial lesions were seen in every case. *Helicobactor pylori* infection was noted in 92%. In non-Hodgkin's lymphoma (*n = *19), 17 cases were of diffuse large B-cell lymphoma, and 1 was peripheral T-cell lymphoma, while 1 was NK-cell lymphoma.

## Introduction

Malignant lesions of the stomach include gastric carcinoma, neuroendocrine tumor (carcinoid tumor), gastrointestinal stromal tumor (GIST), primary malignant lymphoma, malignant peripheral nerve sheath tumor, malignant fibrous histiocytoma, rhabdomyosarcoma, synovial sarcoma, alveolar soft part sarcoma, dendritic cell sarcoma, granulocytic sarcoma, clear cell sarcoma, malignant rhabdoid tumor, choriocarcioma, yolk sac tumor, and metastatic carcinoma [1]. In the present study, 1421 malignant gastric lesions were described.

## Materials and methods

The author reviewed his computer database of gastric specimens detailing 10 000 consecutive gastric specimens taken over the last 12 years 2002–2013 at his pathology laboratory in a relatively large general hospital in Japan. Histological sections were examined when appropriate. Clinical records were also reviewed in the computer system. In appropriate cases, an immunohistochemical study was performed with the use of Dako Envision methods (Dako, Corp, Glustrup, Denmark), as previously described [2–4]. The antibodies employed were as follows: anti-cytokeratin (AE1/3, Dako), anti-cytokeratin (polyclonal wide, Dako), KIT (polyclonal, Dako), PDGFRA (polyclonal, Santa Cruz, CA, USA), CD34 (QBEND10, Dako), vimentin (Vim 3B4, Dako), desmin (D33, Dako), α-smooth muscle actin (1A4, Dako), S100 protein (polyclonal, Dako), p53 protein (DO7, Dako), and Ki-67 antigen (MIB1, Dako). CD3 (M7193, Dako), CD10 (M0727, Dako), CD15 (M0733, Dako), CD30 (M0751, Dako), CD45 (M0855, DAKO), CD45RO (M0834, Dako), CD79α (M7050, Dako), CD56 (MOC-1, Dako), CD57 (HNK-1, Santa Cruz, CA, USA), kappa light chain (polyclonal, Dako), and lamda light chain (polyclonal, Dako). In some cases, Giemsa staining was performed to identify *Helicobactor pylori* bacteria.

A genetic analysis for the *KIT* (exons 9, 11, 13, and 17) and *PDGFRA* genes (exons 12 and 18) was performed in six cases of GIST. The exons of both genes were selected because they are frequent mutation sites in GIST [5–10]. DNA was extracted from the paraffin sections of the GIST and analysed by the PCR-direct sequencing method, as previously described [4, 11–16].

## Results

The gastric specimens were made up of 8579 benign conditions and 1421 malignant lesions. The 1421 malignant lesions resulted from 598 surgeries and 823 biopsies, and comprised gastric carcinoma in 1342 cases (94.4%), GIST in 34 (2.4%), mucosal-associated lymphoid tissue (MALT) lymphoma in 25 (1.8%), non-Hodgkin's malignant lymphoma in 19 (1.3%), and metastatic carcinoma in 1 (0.1%) (Table 1).

**Table 1.** The prevalence of various lesions among 8570 malignant gastric lesions

Malignant lesions	No. of cases (%)
Gastric carcinoma	1342 (94.4%)
Tubular adenocarcinoma	755
Papillary adenocarcinoma	176
Mucinous adenocarcinoma	147
Signet ring cell adenocarcinoma	145
Poorly differentiated adenocarcinoma	114
Adenosquamous carcinoma	4
Gastrointestinal stromal tumor	34 (2.4%)
High-grade	10
Intermediate-grade	16
Low-grade	8
MALT lymphoma	25 (1.8%)
non-Hodgkin's lymphoma	19 (1.3%)
Diffuse large B-cell lymphoma	17
Peripheral T-cell lymphoma	1
NK-cell lymphoma	1
Metastatic carcinoma (from small cell lung carcinoma)	1 (0.1%)

**Figure 1.**
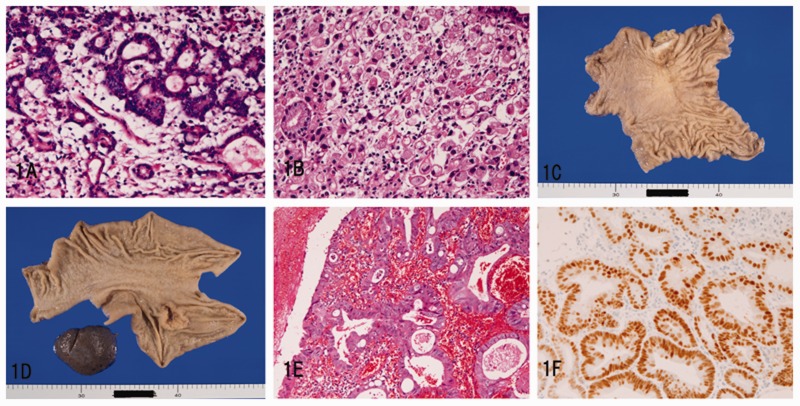
Gastric carcinoma: (A) well differentiated tubular adenocarcinoma (H&E staining; ×100); (B) signet ring cell carcinoma (H&E staining; ×200); (C) macroscopic features of IIc type early gastric carcinoma; (D) macroscopic features of Borrmann III type advanced gastric carcinoma; (E) well differentiated tubular adenocarcinoma arising in foveolar hyperplastic polyp (H&E staining; ×200); (F) tubular carcinoma is positive for p53 protein (immunostaining; ×200)

In the 1342 cases of gastric carcinoma, the histological type according to World Health Authority (WHO) classification was as follows [17]: tubular adenocarcinoma in 755 cases (Figure 1A), papillary adenocarcinoma in 176, mucinous adenocarcinoma in 147, signet ring cell carcinoma in 145 (Figure 1B), poorly differentiated adenocarcinoma in 114, adenosquamous carcinoma in 4, and metastatic small cell carcinoma from the lung in 1. In surgically resected cases, the number of early gastric carcinomas was 258 cases, plus 521 cases of advanced carcinoma. The gross classification of the early carcinoma according to the Japanese Society of Gastric Cancer was Type I 35 cases, Type IIa in 16 cases, Type IIb in 7 cases, Type IIc in 101 cases (Figure 1C), Type IIc + III in 86 cases, and Type III in 13 cases. The gross classification of advanced gastric carcinoma according to the Japanese Society of Gastric Cancer was as follows: Borrmann I in 91 cases, Borrmann II in 193, Borrmann III in 216 (Figure 1D), and Borrmann IV in 21. Twenty-two (1.6%) cases of gastric carcinoma occurred in foveolar hyperplastic polyps (Figure 1E). These carcinomas were well differentiated tubular adenocarcinomas without invasion. *H. pylori* was present in 85% of gastric carcinomas. Immunohistochemically, the gastric carcinoma cells were almost always positive for p53 protein (Figure 1F) and showed high Ki-67 labeling.

**Figure 2.**
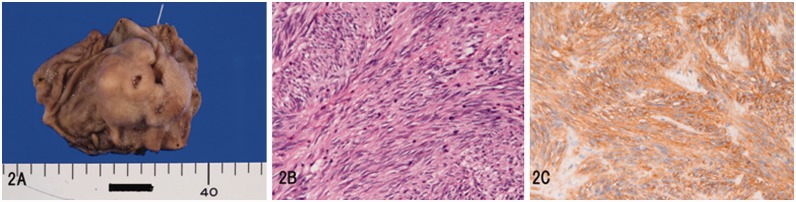
Gastrointestinal stromal tumor (GIST) in the stomach: (A) gross features; (B) spindle cell proliferation is recognizable (H&E staining; ×200); (C) KIT is positive (immunostaining; ×200).

In GIST (*n = *34), tumors were submucosal (Figure 2A) in 28 cases and subserosal in 6. There were 2 cases of epithelioid type and 32 cases of spindle cell type (Figure 2B). The size of GIST ranged from 1–15 cm, with a mean of 5.6 cm. KIT (CD117) was positive in all cases (Figure 2C), CD34 in 31, desmin 2, and S100 4. A genetic analysis was performed in six cases of GIST and showed a total of 6 mutations of exons 8, 11 and 13 of the KIT gene and exon 18 of the PDGFRA gene.

**Figure 3.**
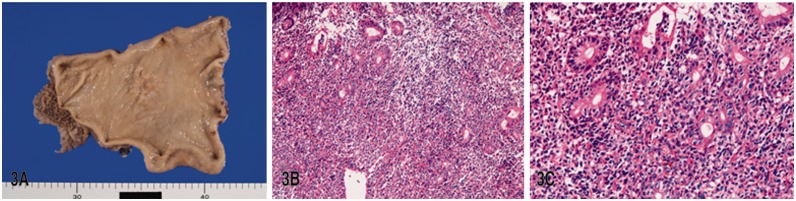
Mucosal associated lymphoid tissue (MALT) lymphoma in the stomach: (A) gross features. (B) proliferation of centrocyte-like cells is visible (H&E staining; ×100); (C) lymphoepithelial lesions are noted (H&E staining; ×200)

In MALT lymphoma (*n = *25), the macroscopic features are indistinguishable from those of early gastric carcinomas (Figure 3A). Histologically, diffuse proliferation of centrocyte-like cells (CCL) (Figure 3B), lymph follicle formation, plasma cell infiltration and lymphoepithelial lesions (LEL) (Figure 3C) were seen in all the cases. *H. pylori* infection was noted in 92%. Immunophenotypes showed positive reactions for CD10, CD20 and CD79α, all of which are B-cell markers. Light chain restriction was present in 18 of the 25 cases.

**Figure 4.**
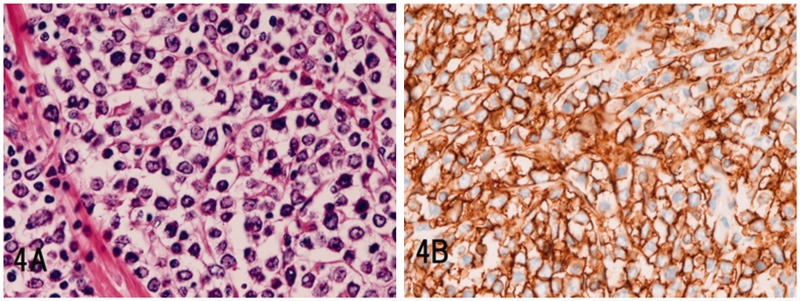
Malignant lymphoma in the stomach (diffuse large B-cell lymphoma): (A) diffuse proliferation of large lymphoma cells is visible (H&E staining; ×400); (B) the tumor cells are positive for CD20 (immunostaining; ×400).

In non-Hodgkin's lymphoma (*n = *19), 17 cases were of diffuse large B-cell lymphoma, positive for CD10, CD20 and CD79α (Figures 4A and 4B), one was peripheral T-cell lymphoma, positive for CD3 and CD45RO, and one was NK-cell lymphoma, positive for CD56 and CD57.

## Discussion

In Japan, the most common malignancy is gastric carcinoma, frequently seen in the present study. In Japan, many persons undergo gastric endoscopy, so that the gastric carcinoma is detected in an early stage. The present study also identified a significant number of early gastric carcinomas. A possible association between gastric cancer and *H. pylori* has recently been proposed. In the present study, the frequency of *H. pylori* was high and it is of interest that 22 early gastric carcinomas were found within gastric foveolar hyperplastic polyps, suggesting that foveolar hyperplastic polyps may be a pre-malignant lesion [18–21].

Our study identified 34 cases of GIST, which is defined as gastrointestinal mesenchymal tumor positive for KIT or CD34 with genetic alterations of *KIT* and *PDGFRA* genes [5–10]. GIST is believed to be derived from interstitial cells of Cajal (ICC) (pacemaker cells) which are present in the muscular layer of the gastrointestinal walls. ICC expresses KIT protein (CD117) and CD34. In practice, immunohistochemical identification of KIT and/or CD34 is a key step in the diagnosis of GIST [5–10]. All types of GIST are considered to have malignant potential [5–10]. According to the consensus report on GIST by Fletcher *et al.* the malignant potential of GIST depends on tumor size and mitotic counts [22].

In the present series, there were 25 cases of MALT lymphoma, which is prevalent in the stomach [23]. This relatively new entity was in the past called reactive lymphoid hyperplasia [23–28]; the condition is often associated with *H. pylori* infection, as was the case in the present study. Eradication of these bacteria often leads to cure of the MALT lymphoma [23–28].

Most gastric lymphomas are of B-cell type [28–31]. The peripheral T-cell lymphoma and NK cell lymphoma are very rare in the stomach. In our study, there were 19 cases of non-Hodgkin's lymphoma; 17 cases were of diffuse large B-cell lymphoma, 1 was peripheral T-cell lymphoma positive, and 1 was NK cell lymphoma.

In summary, this study reported the histopathology of various malignant lesions of the stomach. This report also provided the frequence of the gastric malignant lesions in a Japanese hospital.
